# ArVirInd—a database of arboviral antigenic proteins from the Indian subcontinent

**DOI:** 10.7717/peerj.13851

**Published:** 2022-10-21

**Authors:** Nitin Motilal Atre, Kalichamy Alagarasu, Pratip Shil

**Affiliations:** Bioinformatics, ICMR National Institute of Virology Pune, Pune, Maharashtra, India

**Keywords:** Arboviruses, Antigenic proteins, Epitopes, Indian subcontinent, Database, Dengue, Chikungunya, Japanese encephalitis, West Nile, Chandipura

## Abstract

**Background:**

Studies on antigenic proteins for arboviruses are important for providing diagnostics and vaccine development. India and its neighboring countries have a huge burden of arboviral diseases. Data mining for country-specific sequences from existing bioinformatics databases is cumbersome and time-consuming. This necessitated the development of a database of antigenic proteins from arboviruses isolated from the countries of the Indian subcontinent.

**Methods:**

Arboviral antigenic protein sequences were obtained from the NCBI and other databases. *In silico* antigenic characterization was performed (Epitope predictions) and data was incorporated into the database. The front end was designed and developed using HTML, CSS, and PHP. For the backend of the database, we have used MySQL.

**Results:**

A database, named ArVirInd, is created as a repository of information on curated antigenic proteins. This enlists sequences by country and year of outbreak or origin of the viral strain. For each entry, antigenic information is provided along with functional sites, *etc*. Researchers can search this database by virus/protein name, country, and year of collection (or in combination) as well as peptide search for epitopes. It is available publicly via the Internet at http://www.arvirind.co.in. ArVirInd will be useful in the study of immune informatics, diagnostics, and vaccinology for arboviruses.

## Introduction

With the effects of climate change becoming more prominent, the enhanced propagation of arthropods has resulted in the emergence and re-emergence of Arthropod-borne viruses (arboviruses) worldwide (Ryan et al., 2017; Gould & Higgs, 2009; Mourya et al., 2016). The densely populated South Asian region, geographically known as the Indian subcontinent, with its unique monsoon-driven climate systems, is particularly vulnerable to arboviral diseases such as dengue, chikungunya, Japanese encephalitis, West Nile, Crimean-Congo hemorrhagic fever, Kyasanur Forest disease, *etc*. are on the rise (Nagpal et al., 2016; Dhiman et al., 2010; Shil, 2019; Mourya et al., 2016). In India, between 2015 and 2021, Dengue affected 913,817 individuals (1,490 deaths), Chikungunya affected 82,429 individuals (0 deaths), and Japanease Encephalitis affected 11,283 (1,414 deaths) (NVBDCP https://nvbdcp.gov.in/index.php). Apart from this, there were sporadic outbreaks of West Nile, and Zika. Dengue and Chikungunya are prevalent in all the countries of the Indian subcontinent (Bangladesh (Haider et al., 2021), Pakistan (Islam et al., 2022), Nepal (Rijal et al., 2021), *etc.*). In 2019 Nepal experienced huge epidemic of dengue covering 68 out of 77 districts (Adhikari & Subedi, 2020).

The re-emergence of these diseases is associated with complex factors such as viral recombination and mutation, leading to more virulent and adaptive strains (Tabachnick, 2016; Baylis, 2017). This necessitates analyses of genome and proteome information for tracing viral evolution and phylogenetics, identification of effects of mutations in newly emerging strains, and also the design and development of chimeric peptides for effective diagnostics and/or vaccines. All this research needs basic information about the nucleotide and protein sequence composition and characteristics from viral strains and *in silico* analyses using bioinformatics and immuno-informatics tools.

Since the last decade of the 20th-century emergence of bioinformatics tools, techniques and databases has revolutionized biological research. A huge amount of biological information generated from laboratory experiments is warehoused in databases, which are freely available through the internet. NCBI (https://www.ncbi.nlm.nih.gov/), Protein Information Resource (PIR) (https://proteininformationresource.org/), DDBJ (https://www.ddbj.nig.ac.jp/index-e.html), ExPASy (https://www.expasy.org/), Protein ligand interaction database (PLID) (https://ngdc.cncb.ac.cn/databasecommons/database/id/3794), TTRMDB (http://vit.ac.in/ttrmdb), and dbHDPLS (https://www.dlearningapp.com/web/dbDPLS/index.php) are a few examples of large and popular databases providing information on proteins, protein families and also provide users with online software tools for bioinformatics analyses (Reddy et al., 2008; Zhu et al., 2019; Srinivasan, Natarajan & Rajasekaran, 2020). Bioinformatics databases dedicated to viruses are available. Virus-Host DB is a database that provides users with information on various viral proteins (Virus-Host DB. URL: https://www.genome.jp/virushostdb/). The Virus Pathogen Database and Analyses resource (ViPR) is a valuable resource for viral proteins (https://www.viprbrc.org/). There also exists a database of flaviviruses, FLAVIdB (cvc.dfci.harvard.edu/flavi), which is of limited scope. The most recent dedicated database on dengue is DenvInt (Dey & Mukhopadhyay, 2017).

Bioinformatics plays important role in immunological studies and viral diagnostics like the design of ELISA based tests, chimeric peptides, antigen-antibody interactions, *etc*. (Gangwar et al., 2011; Gangwar et al., 2012; Shil, Chavan & Cherian, 2011a; Shil, Chavan & Cherian, 2011b; Pavitrakar et al., 2018; Pavitrakar et al., 2020) . Hence, quick and easy data retrieval from databases is essential. The practical difficulty faced while searching for Indian sequences is that none of the existing databases enlist entries by country of origin and date of sample collection or outbreak. Most databases enlist sequences by date of publication. So data mining from existing large databases is cumbersome as a large number of entries appears and each had to be read manually to identify the country of origin. Considering the huge burden of arboviral diseases in India and its immediate neighborhood, providing effective diagnostics and phylogenetic studies for tracing viral evolution are important aspects of virology research. Hence, there is a need to develop a database dedicated to arboviral antigenic proteins from strains isolated from the Indian subcontinent.

In this article, we describe the development of a bioinformatics database, ArVirInd, dedicated to arboviral antigenic proteins covering strains reported from India and its neighboring countries. The database is being developed as a knowledge base and envisages providing information on antigenic characteristics apart from the sequence composition.

## Materials & Methods

### Data mining

The antigenic protein sequences for arboviruses isolated from the countries of the Indian sub-continent were retrieved from NCBI. We then searched the internet for the related publication for each sequence and traced out the date of collection or year of the outbreak and the country of origin and information on the host organism (mosquito or human, *etc*.). Searches were made for any available 3D structure for the sequence in PDB. Annotation has been provided for each database entry. The inclusion criterion included complete amino acid sequences for the concerned proteins. Exclusion criterion: all partial amino acid sequences were rejected.

### Data analyses and annotation

For the flaviviruses such as Dengue, West Nile, and Japanese encephalitis the surface glycoprotein (E-protein) and non-structural protein1 (NS1) have been considered for inclusion in the database. For Chikungunya, belonging to the Togaviridae family, the surface proteins E1 and E2 have been considered. The database also includes Chandipura virus envelop protein (G-protein).

Each amino acid sequence was evaluated for antigenic potential using VaxiJen 2.0 server available at http:www.ddg-pharmac.net/vaxijen/VaxiJen/vaxiJen.html (Doytchinova & Flower, 2007). Then the sequences were considered for B-cell epitope prediction using different tools available at the Immune Epitope Database (IEDB) analyses resource (http://www.iedb.org/). Best results from the Kolaskar method of B-cell epitope prediction were considered for inclusion for each entry. The presence of N-linked glycosylation sites on the sequences was checked by using the NetNGly 1.0, a server for N-glycosylation prediction (https://services.healthtech.dtu.dk/service.php?NetNGlyc-1.0).

### Database formulation and architecture

**Figure 1:**
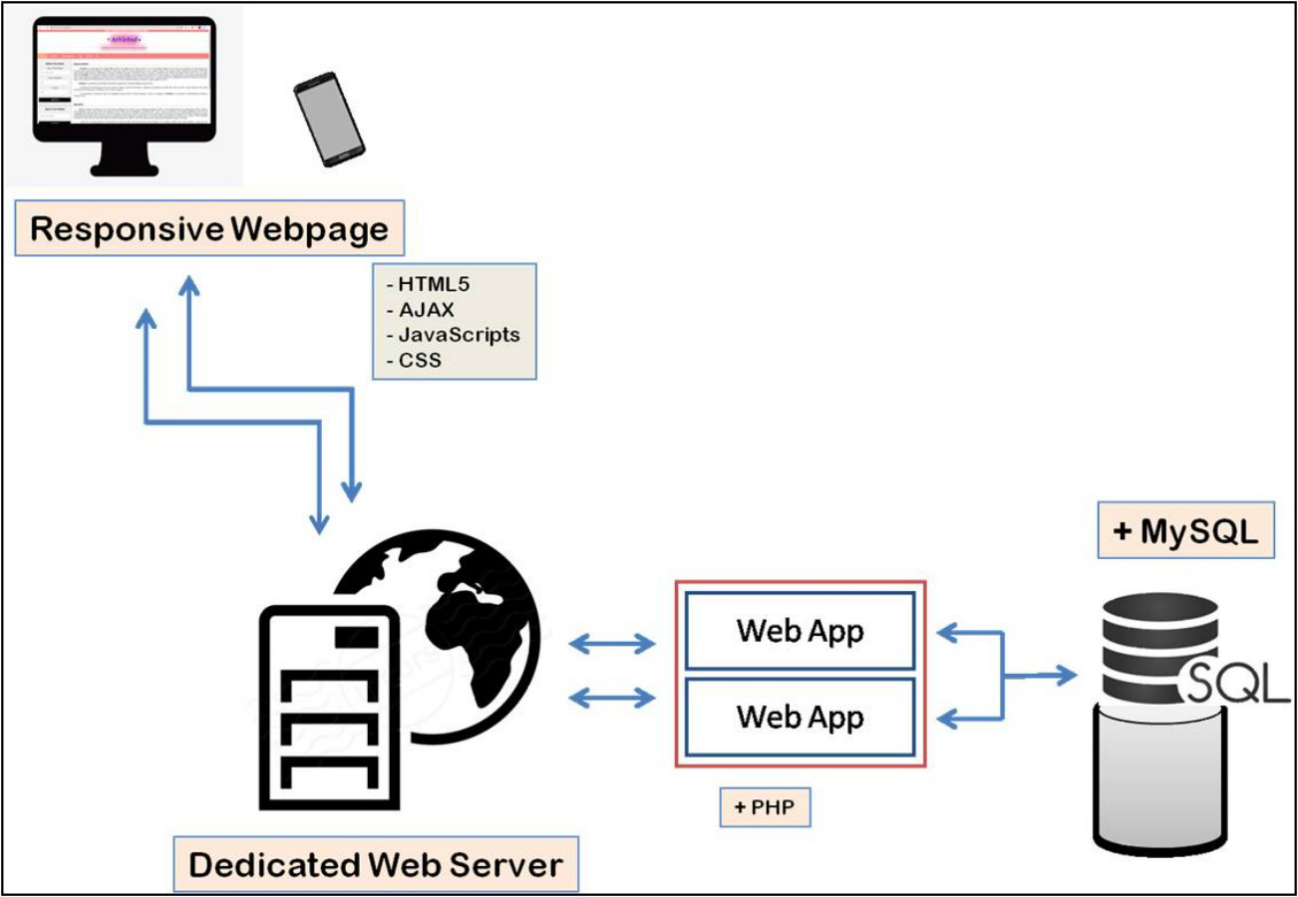
Data processing pipeline for ArVirInd.

ArVirInd has a relational database structure setup using MySQL which facilitates storage, query, and visualization of information. Hyper Text Markup Language (HTML) and Cascading Style Sheet (CSS) have been used for the web-interface design. Annotated sequences data is stored in tables. The application layer between the backend tables and the web interface has been implemented by using PHP. Figure 1 explains the overall architecture of the ArVirInd knowledgebase.

### Dashboard

The ArVirInd dashboard was designed using Google Data Studio (https://datastudio.google.com/) with the connectivity enabled by the ArVirInd MySQL.

## Results

### Database availability and search

The ArVirInd database is open access, annotated, and curated collection of publicly available arboviral antigenic proteins from India and its neighboring countries in the subcontinent. The ArVirInd knowledgebase is available at: http://www.arvirind.co.in.

**Figure 2:**
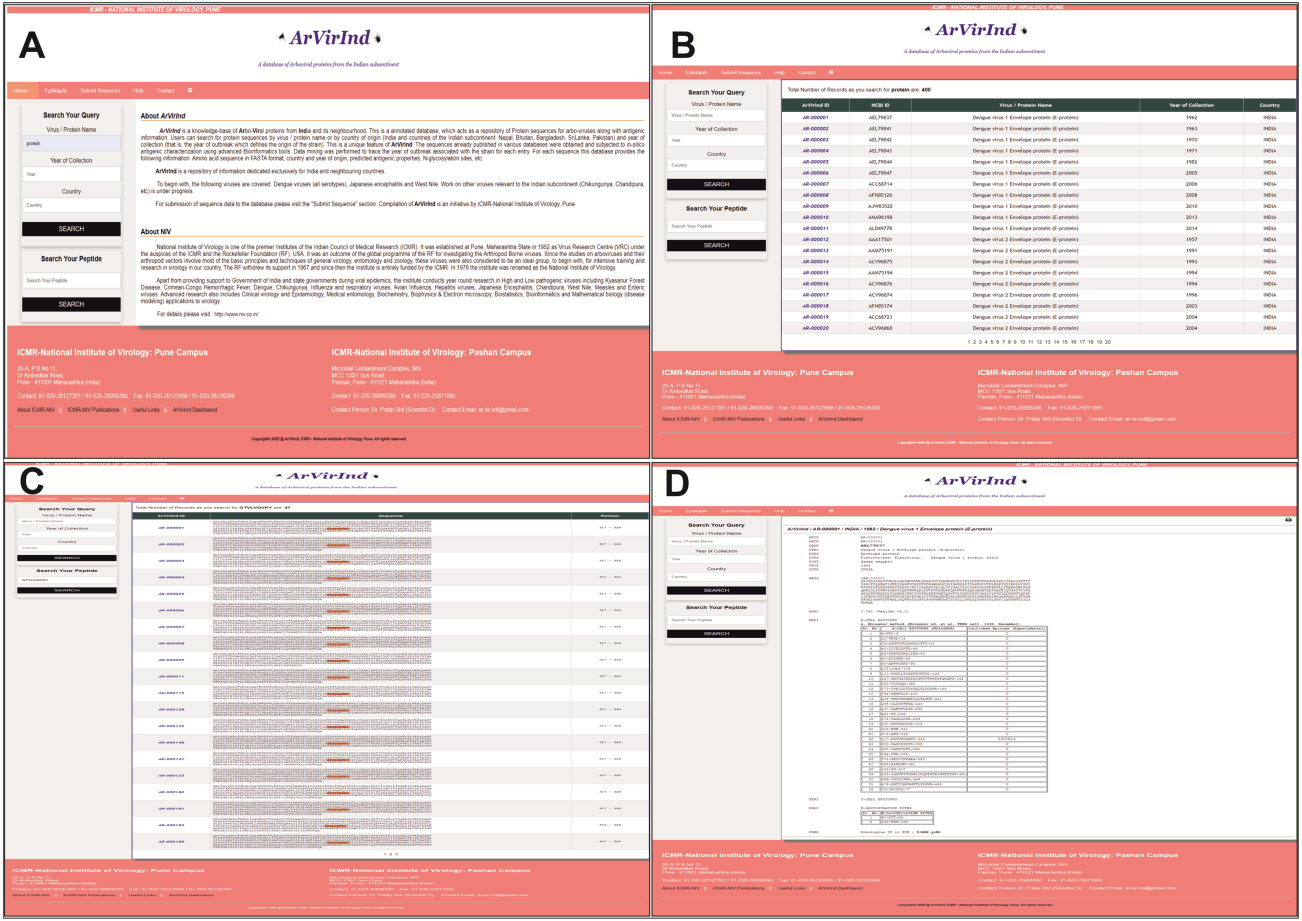
ArVirInd user interfaces: (A) homepage and search, (B) display of search record, (C) result of an epitope search as returned by the ‘Search Your Peptide’ option, (D) database record entry. To search type keywords in relevant fields in the ‘Search your Query’. This returns the list of records available. Clicking on the ArVirInd ID (blue font) will lead to the database record. User can paste the query peptide sequence to search for epitopes using the ‘Search Your Peptide’ option.

ArVirInd homepage provides the user the following ways to search: (a) search by “Virus Name”, (b) search by “Country of Origin”, (c) search by “Year of Origin” or a combination of these options. This is a unique feature of the database. A query returns a tabular output which gives the list of all entries in the searched category. From this table user can choose any particular entry by clicking on the respective “ArVirInd ID”. The record for each entry can be printed or downloaded in PDF format. The display of the ArVirInd homepage is shown in Fig. 2A.

ArVirInd also allows the user to search (Fig. 2B) the database by submitting a query peptide (or epitope) through the “Search Your Peptide” search box (Fig. 2C). AJAX scripting is implemented to search option, which facilitates auto-search and auto-suggest query for the user.

### The database features

Currently, ArVirInd consists of >400 records of antigenic proteins of Dengue, Chikungunya, Japanese encephalitis, West Nile, and Chandipura viruses from the countries: India, Bangladesh, Bhutan, Sri Lanka, Maldives, Nepal, and Pakistan. Users can also search by peptide for B-cell epitopes.

ArVirInd is a unique resource that provides annotated antigenic proteins from arboviruses isolated from the countries of the Indian subcontinent. To start with we concentrated on viral proteins that can generate B-cell and T-cell responses. For example, Dengue virus membrane glycoprotein (E-protein), and non-structural protein 1 (NS1) are known to generate antibodies (Jayathilaka et al., 2018). Record for each entry (sequence) provides information such as unique sequence identifier, accession number, virus and protein name, protein description, source organism, host, year of collection (outbreak), country of origin, the amino acid sequence in FASTA format, the list of B-cell epitopes, antigenic potential (VaxiJen score) (Doytchinova & Flower, 2007), N-glycosylation sites and database cross-references. The details of information fields in the ArVirInd entry record have been provided in the Supplementary Materials. Figure 2D shows the records of an entry in ArVirInd. This database enlists entries by the date (year) of sample collection or outbreak.

At the time of manuscript submission, we had covered strains isolated over 66 years from 1955 to 2021. ArVirInd, being a curated database, relied on complete amino acid sequences as available in the NCBI. To the best of our knowledge, no arboviral antigenic protein sequences were available in the NCBI for India, Nepal, Bhutan, Bangladesh, Sri Lanka, Maldives, and Pakistan for the year 2022 (as the date of sample collection).

### Submission of data to ArVirInd

**Figure 3:**
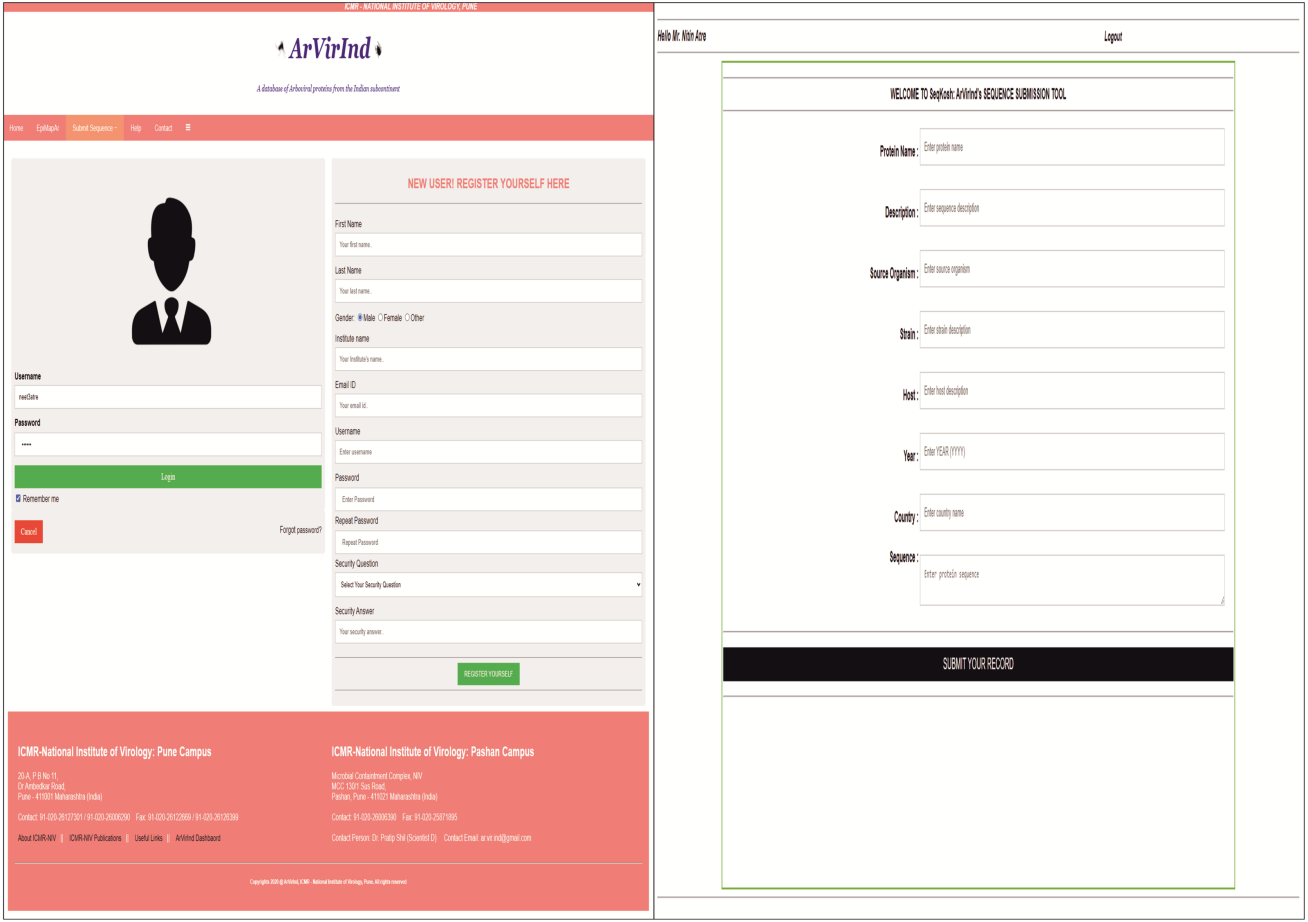
Sequence submission to ArVirInd: (A) User registration; (B) SeqKosh.

The ArVirInd database provides an opportunity for the user to submit sequences of viral strains isolated from emerging outbreaks of Dengue, Chikungunya, West Nile, Japanese encephalitis, and Chandipura. The sequence submission tool is enlisted on the submission page. The user has to register with a valid institutional email id. This will be verified by our team before confirmation of registration. Then the user can login and submit the sequences using our sequence submission tool called, SeqKosh. All user-submitted sequences will be processed, analyzed, annotated, and uploaded by the ArVirInd team. Figure 3 indicates the sequence submission pages.

### Analyses tools

**Figure 4:**
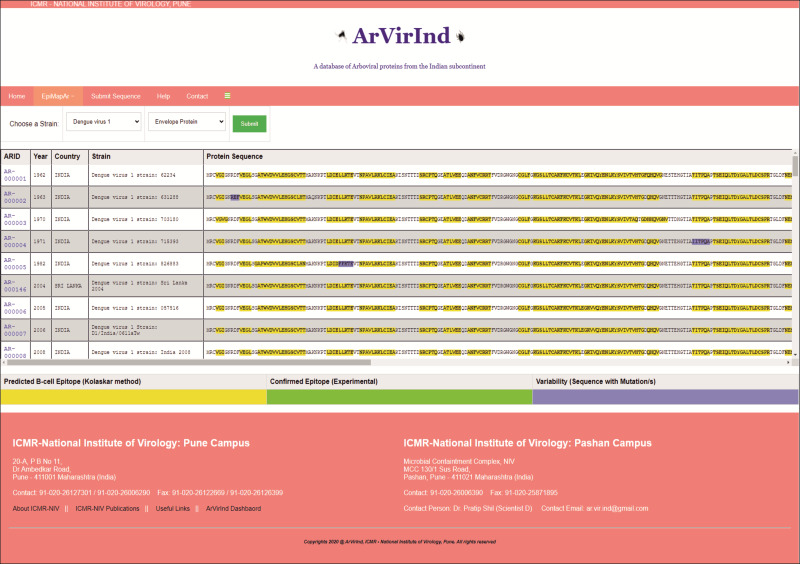
EpiMapAr: a tool for data analyses.

The ArVirInd database has an epitope mapping tool called “EpiMapAr”, which helps display the predicted and known B-cell epitopes (antigenic regions) on the database sequences. This tool can be accessed on the “EpiMapAr” page. This gives the user the choice of “virus” and “antigenic protein”. As an output, it displays all the available sequences in the database for the selected category (Fig. 4).

### Database dashboard

**Figure 5:**
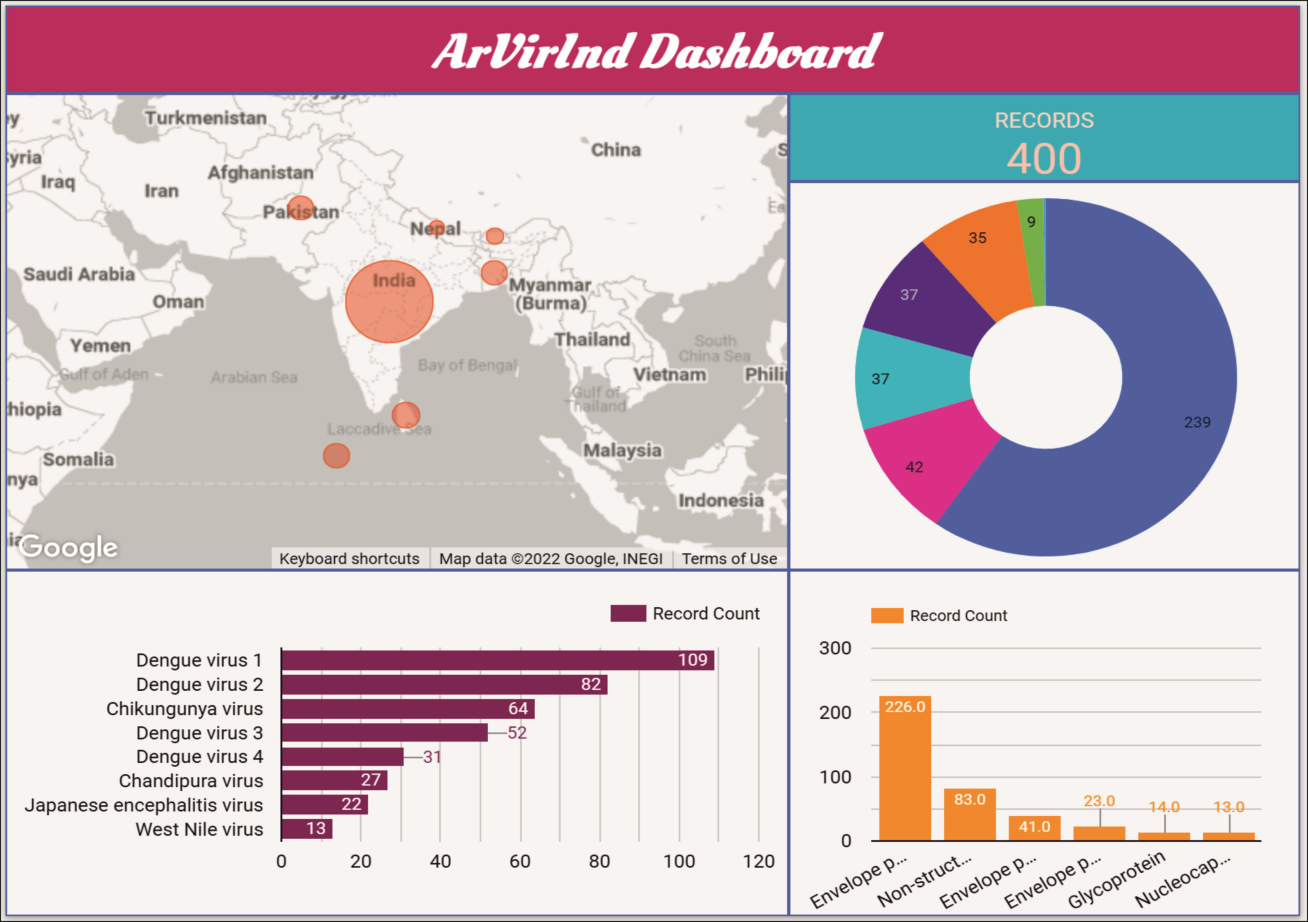
The ArVirInd dashboard.

An interactive dashboard for the ArVirInd database is also available at http://arvirind.co.in/dashboard/. These summaries of the country-wise, virus-wise, and protein-wise records count information about the database (Fig. 5).

### Additional information

The ArVirInd database also provides information about the list of publications of ICMR-NIV, Pune on Dengue, Chikungunya, Japanese Encephalitis, West Nile viruses, and vectors from 1957 to 2020. The ‘important links’ page provides links to other databases and websites important for health sciences and arboviruses. The Help page, describes basic operations for searching, viewing, downloading data, how to use the tools, dashboard, and protocol for sequence submission. The database has been tested for various browser compatibity and was found effiecient for all browsers. We recommend using the Google Chrome browser.

## Discussion

ArVirInd is a first-of-its-kind knowledge base dedicated to antigenic proteins from arboviral strains isolated from India and neighboring countries (the Indian subcontinent). This database enlists sequences by year of collection/isolation which coincided with the year of the outbreak. ArVirInd knowledgebase allows users to search sequences by the year of collection/isolation and country of origin, which is a unique feature. We have carried out exhaustive literature reviews to trace the year of origin for each strain. For each sequence entry, we have carried out *in silico* antigenic characterization using bioinformatics tools. The summary of the findings is available in the database records for each entry. The user can also search by peptide against a record of 8,398 epitopes covering more than 400 amino acid sequences.

The online database also provides the user with an analyses tool. The EpiMapAr tool helps the user in understanding the epitope conservancy between strains (virus-wise) included in the database. This gives the user the choice of “virus” and “antigenic protein”. As an output, it displays all the available sequences in the database for the selected category as below:

– Yellow highlighted sequence pattern represent Predicted B-cell Epitope (Kolaskar method)
– Green highlighted sequence pattern represent Confirmed Epitope (experimentally known).
– Purple highlighted sequence pattern represent Variability (sequence with mutation/s).

The dashboard provides the user with a summary of the datasets. Users can also submit sequences to the database through the “SeqKosh” sequence submission tool.

Dedicated databases exist for epitopes like the Bcipep, AbDb (Ferdous & Martin, 2018; Dunbar et al., 2014) and viruses such as the Ebola virus database (Swetha et al., 2016), or VIPR (Pickett et al., 2012). However, these databases provide specific information about epitopes (known) or pathogens, but none are the repository of strains belonging to specific geographical regions. Finding sequences by country of origin or year of outbreak/isolation from other databases remains an uphill task. We tried to overcome this by creating a database that will enlist sequence information from arboviral strains isolated in the Indian subcontinent region. ArVirInd is user-friendly and enables the user to search for arboviral antigenic proteins by country of origin and year of outbreak/strain isolation. This is a unique feature of ArVirInd. In the future, we will expand the database by including tick-borne viruses such as Kyasanur Forest Disease and Crimean-Congo hemorrhagic fever (CCHF) viruses as well.

## Conclusions

This article presents the formulation of a database of antigenic proteins from arbovirus strains from the countries of the Indian subcontinent. We anticipate that this database will be maintained and constantly expanded with information added for new and emerging strains of arboviruses. We believe that the ArVirInd knowledge base will benefit researchers working in immuno-informatics, arboviral diagnostics, and vaccinology. ArVirInd has the potential to become an important source of information on arboviral proteins for researchers working in India as well as countries like Bhutan, Nepal, Maldives, Bangladesh, Sri Lanka, and Pakistan. The database will be updated and expanded periodically.

##  Supplemental Information

10.7717/peerj.13851/supp-1 Supplemental Information 1 Abbreviations used on the ArVirInd record pages

10.7717/peerj.13851/supp-2 Supplemental Information 2 ArVirInd sequence IDs with the corresponding NCBI IDs

## Additional Information and Declarations

### Data Availability

The following information was supplied regarding data availability: The NCBI sequences used in the database are available in the Supplementary File.
