# Supplementary material for: ArVirInd—a database of arboviral antigenic proteins from the Indian subcontinent
Source: PeerJ. 2022 Oct 21;10:e13851. doi: 10.7717/peerj.13851 (PMC9590419; doi:10.7717/peerj.13851)
Supplement: Supplemental Information 1 [file peerj-10-13851-s001.docx]

**SUPPLEMENTARY MATERIALS**

**Abbreviations used on the ArVirInd record pages:**

| ARID | Unique ArVirInd database ID e.g. AR-000001 |
| --- | --- |
| ARCN | ArVirInd database Accession Number e.g. AR-000001 |
| DBXR | Database cross reference (e.g., [AEL79837](https://www.ncbi.nlm.nih.gov/protein/AEL79837)) |
| VPRO | Virus and Protein Names e.g., Dengue virus 1 Envelope protein (E-protein) |
| PDES | Protein description e.g., Envelope glycoprotein |
| SORG | Source Organism (e.g., Flaviviridae; Flavivirus;    Dengue virus type 1 strain: 62234) |
| HOST | Host from which the strain was isolated. E.g., *Aedes aegypti* |
| YRCL | Year of Collection or outbreak (e.g. 1962, if the year of the sample collection that resulted in virus strain isolation is 1962) |
| CNTR | Country of Origin e.g., INDIA |
| AASQ | Amino Acid sequence in FASTA format. For example:  >AR-000001 MPCVGIGNRDFVEGLSGATWVDVVLEHGSCVTTMAKNKPTLDIELLKTEVTNPAVLRKLCIEAKISNTTTDSRCPTQGEATLVEEQDANFVCRRTFVDRGWGNGCGLFGKGSLLTCAKFKCVTKLEGKIVQYENLKYSVIVTVHTGFQHQVGNESTEHGTIATITPQAPTSEIQLTDYGALTLDCSPRTGLDFNEMVLLTMKEKSWLVHKQWFLDLPLPWTSGASTSQETWNRQDLLVTFKTAHAKKQEVVVLGSQEGAMHTALTGATEIQTSGTTTIFAGHLKSRVEMDKLTLKGMSYVMCTGSFKLEKEVAETQHGTVLVQVKYEGTDAPCKIPFSTQDEKGVTQNGRLITANPIVTDKEKPVNIETEPPFGESYIVVGAGEKALKLSWFKKGSSIGKMFEATARGARRMAILGDTAWDFGSIGGVFTSVGKLIHQVFGTAYGVLFSGVSWTMKIGIGILLTWLGLNSRSTSLSMTCIAVGVTLYLGVMVQA |
| ANSC | Antigenic score based on Vaxijen v2.0 analyses |
| BEPI | List of Predicted B-cell epitopes (based on Kolaskar method) |
| NGLC | List of N-linked glycosylation sites based on analyses using NetNGly1.0 |
| PDBX | Cross-reference with PDB for related protein structure |
